# Beyond ‘health and safety’ – the challenges facing students asked to work outside of their comfort, qualification level or expertise on medical elective placement

**DOI:** 10.1186/s12910-018-0307-0

**Published:** 2018-07-20

**Authors:** Connie Wiskin, Jonathan Dowell, Catherine Hale

**Affiliations:** 1 Interactive Studies Unit, 90 Vincent Drive, Institute of Clinical Sciences, College of Medical and Dental Sciences, University of Birmingham Edgbaston, Birmingham, B15 2TT UK; 2 University of Dundee, Dundee, UK; 3 University of Birmingham, Birmingham, UK

**Keywords:** Electives, International, Ethics, Safety, Students, Evaluations, Supervision, Limitations

## Abstract

**Background:**

On elective students may not always be clear about safeguarding themselves and others. It is important that placements are safe, and ethically grounded. A concern for medical schools is equipping their students for exposure to and response to uncomfortable and/or unfamiliar requests in locations away from home, where their comfort and safety, or that of the patient, may be compromised. This can require legal, ethical, and/or moral reasoning on the part of the student. The goal of this article is to establish what students actually encounter on elective, to inform better preparing students for safe and ethical medical placements. We discuss the implications of our findings, which are arguably applicable to other areas of graduate training, e.g. first medical roles post-qualification.

**Method:**

An anonymised survey exploring clinical and ethical dilemmas on elective was issued across 3 years of returning final year elective medical students. Questions included the prevalence and type of potentially unsafe scenarios encountered, barriers to saying ‘no’ in unsafe situations, perceived differences between resource poor and developed world settings and the degree to which students refused or consented to participation in events outside of the ‘norms’ of their own training experience.

**Results:**

Three hundred seventy-nine students participated. 45% were asked to do something “not permissible” at home. 27% were asked to do something they felt “uncomfortable” with, often an invasive clinical task. Half asked to do something not usually permissible were “comfortable”. 48% felt it more acceptable to bypass guidelines in developing settings. 27% refused an offer outside their experience.

**Conclusion:**

Of interest are reasons for “going along with” uncomfortable invitations, e.g. “emergency”, self-belief in ‘capability’ and being ‘more qualified’ than host-personnel. This “best pair of hands available” merits scrutiny. Adverse scenarios were not exclusive to developing settings. We discuss preparing students for decision-making in new contexts, and address whether ‘home’ processes are too inflexible to prepare students for ‘real’ medical life? Ethical decision-making and communicating reluctance should be included in elective preparation.

## Background

Clinical elective placements are well established as a key and memorable element of training in the UK [14] and other western countries [27]. For the purposes of this paper ‘clinical elective’ is taken to mean an extended placement, in UK curricula typically (but not exclusively) 4–8 weeks duration, outside of the students’ scheduled teaching, self-arranged, often but not always abroad, and forming part of the ‘required progression component’ of their studies. Electives encompass - and are supervised by staff from - all specialties, so relevance is broad. Electives are recognised by the General Medical Council [GMC] in the UK [4], and attract increasing attention as medical schools develop their role in global health teaching [13]. Up to 80% of UK students arrange their elective abroad, with 40% opting for low-resource settings [19, 20].

International electives provide a rich context for learning; not just about new clinical presentations (e.g. tropical medicine) and settings, but affording opportunity for self-development and maturation. Students can consolidate past study, prepare for future challenges (practical and ethical), learn new skills, sense-check career choices [15] and gain a wider appreciation of social and cultural determinants of health, including the global health context (Bateman 2001). Elective participants need to think independently and – e.g. in under-served populations and emotionally difficult contexts – consider their own attitudes and those of others.

However, electives have also attracted criticism, especially given student enthusiasm for experiencing challenging new contexts. Concerns have emerged, e.g., about what students may do when away from the ‘home routine’ (local and therefore familiar processes/environment/support), and the patient safety, student safety and ethical issues associated with such placements [1].

These concerns appear more common for - but are not limited to - resource poor settings. To illustrate the range of challenges, in the authors’ recent experience internal reports have revealed:

- Students being asked to break and reset a child’s fracture without anaesthetic.
- Misunderstandings where a new elective student was mistaken for a new doctor, and expected to operate.
- A student being offered paid locum work prior to qualification because they were considered better than the local staff (within the EU).
- Students initiating life-saving interventions (e.g. successful ventouse extraction) when they were the most experienced person at hand.

However, according to the GMC “The safety of patients and their care must not be put at risk by students’ duties, access to patients and supervision on placement or by the performance, health or conduct of any individual student” [26]. Ramsay and Weijer report that even junior doctors in this environment may be a cause of concern as “Inexperienced surgeons learning surgical skills in developing countries engender greater risk of violating basic ethical principles” [23]. Ethically awkward scenarios can of course be encountered anywhere, and situations students face abroad can - and do - reflect many of the real difficulties and dilemmas newly qualified trainees encounter in their early careers [17].

It is important to understand more fully what experiences our students have working away from their own schools, in order to, e.g., improve visibility for supervising staff and inform pre-departure training and post-elective de-briefing. Schools have a responsibility to prepare students for electives [21], and continue to develop processes for doing so, pre, during and post experience [31]. There is more accumulated literature, however, on threats to their physical health [12, 25, 28–30] than their emotional well-being [18]. There is also a responsibility to the host. To that end the need to promote ethical behaviours on elective is increasingly recognised (e.g. [2, 6]), including resource inequalities between settings [5, 22], but still proportionally little is documented about precisely *what* students are doing ‘away from home’ that might impact – positively or negatively – on both the students and the host’s patients.

The aims of this evaluation were therefore as follows:

1. To establish more fully what students do on different types of elective, including the dilemmas they encounter, how they respond, why they respond as they do.
2. To better understand (building on Dowell’s [7] discussion of pressure on students to “exceed their competence”) why uncomfortable or unfamiliar requests and opportunities are actively taken up, especially where student comfort/safety, or that of the patient, may be compromised.
3. To discover where un-restricted working, or working outside of ‘usual’ undergraduate parameters can occur. The association between ethical risk and exposure to low-resource settings is documented (e.g. [9]), but far less has been reported from developed settings. We also compared student views and experiences across different types of location, which we have not seen reported elsewhere.

The findings may enable schools to make more informed decisions about comprehensive elective preparation, that takes into account the actual reported experiences of a body of students across developed and developing locations.

## Methods

We undertook online surveys in 2012–14 of returning elective students within Birmingham Medical School (between Years 4 and 5) using Survey Monkey. The tool records no personal identifiers or data; for withdrawal a participant would need to contact the administration with the IP of the public/private PC used to submit the form and the precise time of submission. The survey explored their elective activities and views using a mix of single best, multiple, scaled and true/false responses, with additional free-text boxes. The survey was designed with input from a consortium of national elective leads at a routine meeting of the Medical Schools Council Electives Committee. The seventeen leads who attended this particular meeting confirmed the value of the project and refined the questions. The survey was piloted with a year group of students at Dundee as an addition to the routine internal module evaluation to test feasibility prior to the study being undertaken.

Descriptive quantitative analyses utilised MS Excel. Content analysis was used to categorise and interpret free-text responses with categories derived inductively (and independently) by CW and JD. Induction was used as there was no precedent. There were no obvious discrepancies. For a very small number of (minor) matters of interpretation CW and JD met to review the original script, and in 2 cases sought the input of the third author (CH) to confirm interpretation of an ethical term.

The elective experience we evaluated comprised a 4–6 week placement ‘in a healthcare context’, self-organised by the student. Support for this module is from a home supervisor (to facilitate planning and preparation pre-elective, and rate and de-brief the experience post-elective), and a location supervisor (a named individual at the host site, who commits to the day-to-day oversight of the placement, and reports back on professionalism). Preparation includes introductory and pre-departure lectures, access to a post-exposure prophylaxis and infectious disease service, talks from doctors who have worked extensively in developing settings, presentations from previous prize winning students, and an evening road-show delivered by the returned student year (featuring indemnity and insurance providers, travel advisors, ethical electives representatives, and students with first-hand experience of electives in each continent). The module is assessed, specifically a detailed protocol, health and safety review and risk assessment in Year 4, and 3000 word academic report in Year 5. Protocols are reviewed by both the home supervisor and an Elective Committee (second screening) for feasibility and safety pre-departure. Students define whether their setting is developed or developing in their risk assessment, using, typically, WHO categorisation.

Data were collected from returning elective students 2012–14. An initial email link was sent, as is standard module evaluation practice, with a 2 week follow up for each cohort.

This project was an educational evaluation (part of routine module feedback), approved at The College of Medical and Dental Sciences, and as such did not require external approval. Students are consented to receive and take part in course evaluation for all progression modules. The content was authorised by the (then) Vice Dean for Education, and students were informed in writing that the purpose of this particular evaluation was, in addition to influencing next year’s teaching, to better understand student experiences away from home, and inform on-going evaluative research. Completion was not mandatory, and participants were informed that the software cannot identify any individual information (the only means of identification would be if the student disclosed to the administrator the exact time of submission and IP address of the computer used).

## Results

### Demographics

A total of 379 out of 768 students completed the survey (response rate 49%). Respondents were 68% female (255); 38% (144) were from ethnic minority groups and 4% (15) were international students (broadly consistent with overall cohort demographic of 64% female: 36% male ratio and 5% international). Most quantitative questions were completed by all responders, but any missing are indicated. Free-text comments were invited where explanation was desired, and so response varied.

### Location and supervision

Thirteen percent (50) stayed in the UK, 27% (103) visited another developed country and 56% (214) a developing country. Two percent (6) were international students ‘returning home’, with the remaining 2% reporting combined experiences.

City locations – from response rate 362 - were prevalent at 65% (compared to town or rural), and most students were supervised by a hospital consultant (56%; 202), registrar (18%; 66), or equivalent, compared to 5% (18) working with a General Practitioner. The remainder reported a range of supervisors, including Primary Care staff, aid workers, nurses, junior doctors, and allied health professionals.

### Frequency of requests not expected in UK undergraduate training

Just under half said they were asked at least once (Table 1) to work outside of their usual parameters. Nine (2%) reported this being “all the time”. We did not detect a relationship between host supervisor seniority/specialty and the likelihood of being asked to do a task not expected in the UK.

**Table 1:** With what frequency were you asked to do things not typically asked of or permissible for a UK undergraduate?

Frequency	Response % (frequency)
Never	55.09% (205)
Once	7.39% (28)
A few times	29.02% (110)
Regularly	8.12% (27)
All the time	2.37% (9)

### Knowledge of, and confidence in, what is permitted

Most (341; 91% see Table 2) claimed that they were confident that they knew what they should and should not be asked or permitted to do at their current level of undergraduate training but 39% (147) had been asked or allowed to do something not condoned in the UK.

**Table 2:** How true where the following statements for you - thinking about your professional experience (i.e. not social or leisure) on your placement?

Statement	True	False
On Elective I was never asked/allowed to do anything that I wouldn’t have been asked/allowed to do as a UK undergraduate medical student	61.11% (231)	38.89% (147)
I am confident that I know what I should and should not be asked/instructed to do at this stage in my training	90.69% (341)	9.31% (35)
I am confident that I know what I should and should not be allowed to do at this stage in my training	90.16% (339)	9.84% (37)
If I was asked to do something, and wasn’t sure if I should, there was always someone to help clarify	76.33% (287)	23.67% (89)

### Comfort level, and saying “no”

While 27% of students (104) were asked on placement to do something they felt “uncomfortable” being asked to do, most - 73% (275) - were not. Of those who were asked to do something “uncomfortable” 14% (53) reported the discomfort relating to an “invasive clinical task”, 13% (48) a “non-invasive clinical task”, 6% (22) a “non-clinical task” and 4% (17) an “ethical decision” (Some students experienced multiple factors). Six were “unsure”.

In a question specifically about saying “no” 71% (268) reported “I wasn’t asked to do anything that **I considered** unusual or unsafe, so refusing didn’t come up”.

In a separate question, 27% (103 students) reported having refused to do something outside their UK parameters. Only five respondents said “yes” to something which on reflection they wished they had not.

The 78 free-text responses about working out of usual limits resisted systematic categorisation, as they related to descriptions of incidents unique to the individual site and student. However, broad themes were encouraging, with 18 respondents describing refusals, and 29 reporting how they explained their need for guidance or supervision before undertaking the unfamiliar. However, two were potentially concerning responses, e.g. “Why wait 2-3 years if you can do it now”.

### Permissibility and acceptability

In responses about when working beyond usual home institution parameters is ‘permissible’ (Table 3), “clinical emergency” was the most frequently selected justification for working beyond home norms. The wishes or invitation of the host were identified as justification by considerably fewer than the selection rates relating to students seeing themselves as the ‘most qualified’ present or “knowing their capabilities” regardless of not having yet passed professional examinations. Free-text comments linked to this question primarily added the caveat of the requirement of appropriate supervision (over half directly referred to this), but some related to communication, e.g. reluctance to challenge a “senior”, worry about causing “offence” and problems finding a way to initiate discussion about expectations and personal reservations. The 19% (69) who said the chance of atypical working had informed their choice of placement merits further consideration, as picked up in the Discussion.

**Table 3:** When it is permissible on Elective to work outside your level of undergraduate experience?

When the host tells you to (instructional)	14.25% (52)
When the host asks you to (requests)	13.42% (49)
When you know you are capable, irrespective of whether you’ve had a chance to do the relevant exam yet	51.51% (188)
When that direct experience/responsibility is why you chose the placement in the first place	18.90 (69)
When you are the most qualified person available in the moment	57.53% (210)
In clinical emergencies	71.51% (261)
To be perceived as being helpful	3.84% (14)
When local medical students are allowed to do the things you are being asked to do	21.64% (79_
Other	7.40% (27)
Total Respondents: 365 (multiple answers accepted for individuals)	949

### Acceptability in developing world setting

#### A quantitative responses

Only 3 respondents felt students were ***not*** more likely to be asked to work outside UK parameters in developing – as opposed to developed – countries (Table 4). Just under half (49%) believed it “more acceptable” to work outside UK parameters in developing settings. 21% (78) were “unsure”.

**Table 4:** Likelihood and acceptability of working outside UK parameters in developing world settings

Question	Yes	No	Unsure
“Do you think students are more likely to be asked to work outside of their ‘normal UK parameters’ in developing countries than in developed countries?”	88.36% (334)	0.79% (3)	10.85% (41)
“Do you think it’s more acceptable for students to work outside ‘normal UK parameters’ when in working in developing countries?”	48.93% (183)	30.21% (113)	20.86% (78)

#### B qualitative responses

Picking up on findings above (7A), 164 respondents provided additional text comments, from which 12 themes emerged, ranked below by frequency of reference:

1. Resourcing issues (including lack of more experienced staff) (63)
2. Degree of supervision available (21)
3. It (working outside norms) is not acceptable (20)
4. Flexible approach required, but with safety in mind (19)
5. Medical ethics (16) - 5 ‘litigation’
6. Work to the cultural norms of the host student (16)
7. Benefit to the learner (11)
8. Patient and staff acceptability (7)
9. UK parameters considered too restrictive (7)
10. React ‘in the moment’ (4)
11. Insurance (3)
12. Geographical location (2)

These results are displayed ranked by frequency of response, but reflect both reasons ‘for’ and ‘against’ a flexible approach to working outside their training norms. Results reflecting arguments against working outside of norms are ***3rd****,* ***5th and 11th*** on the list. These are in a minority, and relate to ethics and legality rather than competency. ‘Resourcing [limitation]’ was the top answer, with comments including “I was the most qualified person there”, “I actually knew more than the resident staff”, “I was better than nothing”, “I was the only person available” and so on. Point 9 relates to curriculum restrictions and preparation for postgraduate practice. As one respondent fed back “Many procedures could be considered ‘risky’ but are performed regularly by practitioners. These skills must be learnt and we are privileged to have the elective opportunity to practise these skills with enthusiastic doctors/nurses”.

### Harm and risk

Experience of a personal “adverse event” was reported by 52 (14%) students. These included needle-stick injury, infection risk, accommodation/transport problems, emotional events such as loss of patient(s), crime/intimidation and personal illness.

In relation to patient safety 19 (5%) answered “Yes” to the question *Did you personally take part in anything unusual that could - potentially - have put a patient at risk?* However almost double that number, 36 (9%), answered “yes” to *Did you take part in anything unusual where if you hadn’t got involved a patient could have been at risk*?

All of those who had put a patient at risk offered a description of the event. One case related to an insurance fraud, another to overly relaxed standards that might have contributed to a neo-natal death. The remainder described clinical procedures and responsibility level (“running ward rounds”; “working unsupervised”). Comments describing scenarios where *refusal* to participate might have impacted on patient safety related to acute incident (“cardiac arrest” featured 4 times), the student noticing something that other staff had missed, or birthing. Two students intervened on prescribing errors.

Adverse events (response rate 377) were present across locations; more prevalent in, but not exclusive to, developing world settings. Of the events reported (14% of respondents; 53 students) 21% of these reported risk exposure in a developing setting, 5% in another developed (non UK) setting, and 3% in the UK. Adverse event descriptions included ethical themes, e.g. “lack of privacy” and “(psychiatric) physical restraint of a patient”.

### Variation in student response between developed world and developing world settings

Sub-analysis was undertaken to look at the relationship between setting – UK, other developed, or developing – and response given across fields (Table 5). This included the finding that student reported confidence in knowing what they should or should not be asked/allowed to do was lower for students returning from developing than developed settings, although by a relatively modest difference of 6.1%.

**Table 5:** Comparison of results between developed and developing settings

Response	Yes	Yes	Yes
Summary questions	UK setting	Other developed setting	Developing setting
On Elective I was asked/allowed to do something that I wouldn’t have been asked/allowed to do as a UK undergraduate medical student	11.4%	20.5%	55.8%
If I was asked to do something, and wasn’t sure if I should, there was always someone to help clarify	94.1%	93.2%	65.7%
I think it more acceptable for students to work outside ‘normal UK parameters’ when in working in developing countries?	37.1%	47.2%	53.8%
I think students are more likely to be asked to work outside of their ‘normal UK parameters’ in developing countries than in developed countries?	71.4%	89%	95.3%
I was asked on placement to do something that I felt uncomfortable with, in terms of my current level of qualification?	2.9%	6.8%	20.9%
I took part in something unusual that could - potentially - have put a patient at risk	0%	1.4%	7%
I took part in something unusual where if I hadn’t got involved a patient could have been at risk	2.9%	4.1%	15.8%

The elective location appeared to influence student perceptions of the acceptability of working outside of UK parameters, but difference here was not marked. 37.1% from UK settings, 47.2% from other developed settings and 53.8% from developing settings felt it was more acceptable to work outside qualification level in developing countries.

The highest proportion of within-group experiences of discomfort were reported from developing settings (20.9%); but not exclusively with 2.9% of UK based students and 6.8% of students in other developed settings reporting this. Managing working outside of expectations was not exclusive to poorer environments; two UK based students and 6 non-UK ‘other developed setting’ students were asked to do an invasive clinical task that they were uncomfortable with, compared to 37 in developing settings. Descriptive examples of uncomfortable tasks (i.e. additional text box comment) almost all came from developing world attachments. Examples of student reported instances of working outside of UK training level expectations are in Appendix 1.

## Discussion

To our knowledge this is one of the largest surveys of its type, and the only one comparing acceptability perceptions across settings (Results 5). Key findings confirm – aligned with other literature - that students continue to have opportunities to work outside their ‘home’ parameters while on elective, but of note here that this phenomenon is not exclusive to developing world settings. As expected, reported levels of unrestricted opportunity were higher outside of developed settings, but examples came from all settings, including the UK. Focus on elective preparation can tend towards the risks associated with new, remote and unfamiliar environments. This paper is a reminder that risk is everywhere, and students are not immune from ethical dilemmas and difficult decision making just because the setting is geographically (or culturally) ‘close to home’. This can usefully inform that way pre-departure risk assessment is conducted, including, e.g., “saying no” [2] and maintaining standards in a home as well as international setting. While the paper confirms reports in other literature about the type and range of ‘new’ activity students may be encouraged, or expected, to participate in, an addition is the emphasis respondents here placed on availability and desirability of supervision. Much reported here has resonance for ‘first day of work’. Student perception is that applying UK training standards abroad is helpful – they reported contributing positively to the standard of care twice as often as they reported things not learned in the UK. This positive contribution has not been studied or described in detail and anecdotally contributes significantly to learning; “For the first time I knew why I was studying medicine”. Perhaps students can both contribute positively in lower resource settings and learn from appropriate valuable experiences. However, while valuing student response, more work needs to be done to contextualise how their assessment maps with, eg, host perceptions [16].

The points below reflect the papers aims of exploring (1) “what”, (2) “why” and (3) “where”.

1. Findings did confirm increased frequency of exposure to unsupervised working, unusual scenarios and adverse events in developing locations, but the overall risk of harm to patients was reportedly low and possibly outweighed by instances of protecting patients [Results 8]. Twice as many felt they had improved rather than jeopardised care when out of their comfort zone. Of course both are student perceptions. The cultural context they were in or resource issues they experienced may be of relevance to how they defined their ability to contribute, and their hosts might take a different view of their effectiveness. However, findings here potentially counter more alarmist reports, so future studies might explore positive impact and the degree to which responsible students pro-actively seek guidance. The students’ management of the opportunity is, perhaps, as important as the opportunity having being offered.
2. An emerging theme was that while students did agree to participate in unusual activity (in line with what is previously known) there was – interestingly - a reported level of comfort associated with this. Students (91% Results 2) report “knowing” what is and is not permissible, but half of the cohort was “comfortable” with being asked to do something that would not be typical at home. This challenges us to re-consider what is ‘allowed’ in the UK. Permissible activity may be more influenced by teaching logistics and procedure than by what is acceptable, or what the student is actually capable of. A UK student on elective in any setting (including home) may well have opportunities outside of ‘the norm’ simply because the specialist nature of many electives and supervision arrangements make these opportunities feasible. The data do not allow us to precisely determine the true motivations of the 19% (Table 3) who reported the potential to work outside their normal parameters as a driver for that placement choice, but there are two perspectives. We might assume them reckless, and intent on risk-taking, but equally they could have recognised gaps/deficiencies on their taught course and sought appropriate supervised experiences to address them.

Knowing what is and is not allowed - for which confidence was high - therefore does not necessarily translate into discomfort. Students may or may not be astute judges of their own knowledge and ability, but they can arguably be reasonable judges – in the moment - of their own comfort. This could suggest coping rather than being maverick (Results 7b.9), raising the question of the degree to which the contemporary teaching hospital environment and high student numbers can ration clinical learning opportunities. An emergent question is the degree to which reportedly felt comfort is warranted, which could vary depending on the reflective ability of the individual, but remains a potential area for future scrutinty involving student and staff stakeholders.

Confirmation that students were invited or given scope to work outside their expertise was anticipated, so more novel were the reasons students reported for going along with such invitations. Resisting someone “senior” (Results 6) is challenging. This is not just an ‘elective issue’ but highly relevant to home students and graduates across the NHS, as highlighted in The Francis Report [11]. Undergraduate communication training tends to focus on core established fields; breaking bad news, history-taking, shared decision making, resolving conflict and so on [10]. While these are important, there is a prerogative going forward to equip tomorrow’s doctors with the confidence and skill to know when - and how - to ‘say no’, and how to highlight problems. Helping learners to develop resilience and gain confidence in raising concerns are educational priorities. However, the result that 27% (103) *had* refused to do something outside their limits is encouraging, suggesting that some students can, and do, decline.

In addition, students’ uncertainty about ethical parameters, lack of experience in ethical/moral decision making and weighing up ‘help vs. harm’ arguments (including utilitarian and deontological viewpoints) need consideration. Students may be familiar with the theories, but applying them when supervision is limited and/or the context unfamiliar is another matter. The dilemma when a student in any scenario is asked to do something unusual/unexpected is complex, involving degrees of legal, ethical and moral reasoning (potentially exacerbated by a new cultural context). Case studies have, usefully, been reported elsewhere [8]. Our findings highlight the importance of access to supervision and in some instances peer support, but more work is needed to understand this, and how, such dilemmas are actually resolved. The law and professional ethics codes clarify what has to be done, or not done, to avoid legal or professional penalties, while moral codes often have less prescriptive sanctions, rendering decisions more a matter of personal conscience. Outside of the UK both areas – legal/ethical frameworks and moral obligation, may be less clear or familiar to students, leading to high challenge but also to learning opportunity. Medical educators might consider how to address and optimise this within elective planning, preparation and debriefing.

A dominant narrative from developing world settings was, predictably, that of ‘the best available hands’. This is distinct from the emergency situation where - when no-one else is available - a senior student could be expected to intervene [3]. It refers instead to the learner’s perception that s/he was the “best” option for the patient, irrespective of whether s/he was qualified by home standards in that intervention. This manifested repeatedly, with explanations for working beyond limitations often relating to resource poverty (Results 7b). At one level intervention under such circumstances may be entirely reasonable, but the students’ predicament is not completely circumstantial, nor beyond their or their medical school’s control. This ‘best available’ scenario is arguably predictable (the student has after all *chosen* a remote or under-resourced setting) and so seems to merit further consideration, mindful that, at least in students’ eyes, they are typically doing more good than harm.

3. The emergence of examples of dilemmas from home and other developed settings is potentially important. 8% reported an adverse event in a UK or other developed setting; that is 30 separate students. 40% of respondents in this survey overall did their elective in a developed setting, and numbers of students within this group (11% UK and 20% other developed location) were asked or invited to work outside of their limitations. Supervision here is more easily accessed, but the exposure to the offer remains noteworthy. It is possible that as financial burden on students increases, and electives become increasing viewed as career tasters, that ‘home’ and ‘developed’ experiences will increase in frequency.

Perhaps the overall issue is not so much about re-visiting worries about loss of supervision and the nature of independent clinical work on (any) elective, but reflecting on the degree to which students are restricted from experience in their home institutions. For some students freedom is central to the appeal of electives, and it may be that students feel that rationed clinical experience towards the end of their degree does not fully prepare them for decision-making and working life. Students in many countries are very involved in patient care (e.g. North America) or learn an extensive range of direct practical skills (e.g. South Africa), so restrictions based on custom rather than data about harm and benefit merits review. That is not to say that students should have un-regulated access to patients in any location (patient and public safety remains, of course, paramount), but we might listen more, as we have found here, when they tell us what electives add that existing curricula lack.

Formalised preparation for multiple sites implies consistency, but rationales exist for and against care standards for electives. Maintaining principles while resisting cultural imperialism is a sensitive and contentious subject – challenging for the seasoned clinician let alone the undergraduate. Would an international student on elective in the UK be welcome, e.g., to apply their ‘norms’ rather than adhere to local expectations?

### Limitations, and developments

This study presents data from a single UK school; however anecdotal evidence from national Medical Schools Council Elective Commitee meetings and presentation of results suggests widespread shared concerns. Improvements could be made, including the separating out of question wording more precisely in future iterations (ask and allow, e.g., are not inter-changeable). The evaluation achieved a reasonable response rate, but response bias, variance in student interpretation or potential reluctance to disclose concerning activities/views (social acceptability bias) could have influenced outcomes.

Use of free-text response enabled a degree of categorisation of results and the type comparison of responses by elective location shown in Table 5 (confirming differences between settings) is infrequent in the literature. This approach could be reproduced on a larger scale so multicentre or international studies are sought going forward. Qualitative analysis of data in the survey relating to student definitions of ‘ethical elective’ is underway, to be published separately.

## Conclusion

Improved understanding of the student experience on elective, and the reasons they cite for making the decisions they do, will aid pre-departure preparation. Attempting to prepare students for every ethical scenario across the globe would be futile, as would be claiming to prepare them for every conceivable scenario that could occur on their first day of qualification. However, offering more robust, generic training on ethical decision-making and the effective communication of personal anxieties and limitations is achievable. These skills would also inform good practice in other situations where junior staff can feel out of their depth. As a result of this, and other, evaluations a new teaching session has been implemented in Year 4 on ‘raising concerns’ and pre-elective departure training now includes more on ethical considerations.

The frequency and diversity of situations reported raises questions about student conduct, and preparing them – to the best of our ability – for a breadth of settings. The need to address professionalism through teaching is not new. As Shah and Wu [24] surmised: “While medical training programmes are beginning to provide students with the knowledge to put their global health experiences in context, they must remember that they also bear the responsibility of training their students in a framework to approach these experiences in a principled and professional way. It is necessary that these institutions provide adequate and formalised preparation for both clinical *and* ethical challenges of working in resource-poor settings.”

Thus, the issue becomes one of managing expectations (both student and host) and of sound educational governance. Self-directed electives may not be appropriate for all students in all environments, but students may continue to arrange them, underscoring the need for clearer frameworks to benefit all parties. Unless we gain increased understanding of what happens at the destinations students choose it is difficult to offer appropriate preparation, and without links, feedback or partnership of some sort there is reduced hope of influencing, or understanding, host expectations.

## Appendix 1

Examples of student reported instances of opportunities to working outside of usual UK parameters

### Refusals in developed setting

*[I] was asked to scrub in and help with surgery, and refused.*

*In the first few days of elective, I said no to some of the things other medical students were expected* *to* *do including 24 h on call shifts.*

*This was related to giving Vit[amin] C to a patient with Cancer as she had been told it would cure her tumour. I refused on ethical ground.*

### Refusals in developing setting

*I refused to sign prescriptions even though i [*sic*.] was writing them, but the doctors were happy with me signing them.*

*Skin grafting - obtaining the skin graft, I felt I was no way near trained enough to do this, and in addition I was feeling unwell at the time and thought it would be irresponsible to attempt such an advanced procedure when I knew my concentration level was lower.*

*I said no to taking blood because of the high prevalence of Hep B, and no to examining an unisolated [*sic*.] TB patient.*

### Opportunities where student felt uncomfortable developed setting

*I was asked to stitch up a man’s lip, which I had not done on a human before, but we had received training on suturing at the start of our placement and before starting the procedure, I spoke to the supervising doctor about the process to refresh my knowledge. I did not feel uncomfortable as such, but nervous as it was my first time.*

*I wa[s] approached by a patient in acute distress, however I had no training in how to deal with this situation, but it was expected of me to have a full working knowledge.*

*[I] was told to conduct a flexible cystoscopy on a female patient.*

### Opportunities where student felt uncomfortable developing setting

*I assisted C-sections which were much more involved than in UK. I explained my lack of expertise but was willing to learn within limits/if they were comfortable. They were so short staffed, however I had to assist and learnt the hard way.*

*Suturing in A&E, first time doing sutures and without supervision. Had seen one before so was able to complete the procedure.*

*I was frequently asked to take blood from children, which I am not trained to do.*

*In XXX [country anonymised] when refusing to rebreak a child’s arm without anaesthetic – was told “you should learn not to be such a sentimental XXX [gender reference]”!*

### Situations not usually permissible for students in UK that could have exposed patient to harm (none offered from developed setting)

*In theory, doing the ward round by myself could have put the patients at risk. But i[*sic*.] was very conscientious in making sure I got a second opinion where needed and even went in out of hours to check on patients I was concerned about. I do not feel any patients were put at risk by me doing the ward round. On [*sic*] the contrary, I believe that they actually received more 1–1 time in history and examinations and that I was more thorough than the local doctors (who often asked me for advice!)*

*The neonatal care was very poor. Often they recussitated [*sic*.] a new born using nasal specs instead of a mask and high flow oxygen. We saw babies die and mothers bleed to death because there seemed to lack a sense of urgency.*

*One occasion of prescribing not under supervision - there was no doctor available and a child was admitted with severe malaria anaemic and hypovolaemic. I prescribed iv [*sic*] quinine which was later cancelled due to the risk of fluid overload.*

### Situations not usually permissible for students in UK that helped avoid exposing patient to harm

*Patient with history of mental health problems was convinced by the locations team that dietary changes could solve her condtition [*sic*] and she did not need to take her medication. [I]convinced parents of the minor to seek proper medical attention (UK).*
*I recognised a child with a blocked airway and I decided to act on this without asking permission from other staff there (developing setting).*
*[*sic*]resuscitation and oxygen therapy of a newborn after c-section when all other medical staff were busy caring for the mother (developing setting).*

## Abbreviations

CW: Connie Wiskin
GMC: General Medical Council
JD: Jon Dowell
UK: United Kingdom
WHO: World Health Organisation
